# Development and initial psychometric properties of a panic buying scale during COVID-19 pandemic

**DOI:** 10.1016/j.heliyon.2020.e04746

**Published:** 2020-09-02

**Authors:** Samuel Lins, Sibele Aquino

**Affiliations:** a University of Porto, Portugal; b Pontifical Catholic University of Rio de Janeiro, Brazil

**Keywords:** Psychology, Fear, Panic buying, Psychometric properties, Consumer behavior, COVID-19, Consumption, Validity, Reliability

## Abstract

Fear is a powerful driver of human behavior, even more during times of crisis. Panic buying occurs when fear and panic influence behavior leading people to buy more things than usual. So far, no specific scale on this has been found in the major databases, thus the aim of this exploratory study is to develop a Panic Buying Scale (PBS) during COVID-19 pandemic. 393 Brazilians took part in this study (251 women and 142 men), answering a sociodemographic questionnaire and instruments of these variables: (1)panic buying, (2)impulse buying, (3)temporal focus, (4)optimism, (5)risk perception, (6)need for cognition. Data collection was conducted through an online questionnaire which was shared through social media networks, from April 10th to May 4th, 2020. Factorial exploratory and confirmatory analysis indicated that PBS has a unidimensional solution and showed satisfactory reliability indexes. Results revealed that men buy more by panic than women. PBS also was positively correlated with impulse buying, past and future temporal focus, and risk perception; as well as negatively correlated with optimism and age. Findings suggest that PBS is psychometrically acceptable in the Brazilian context. This new instrument can be useful to understand the psychosocial phenomena associated with consumer behavior. Future investigations could provide more evidences of validity in other contexts.

## 1 Introduction

It is unquestionable the impact of COVID-19 pandemic in all domains of our lives. All these uncertain times evoked deeply negative emotions, like fear and panic. In fact, fear is a powerful driver of human behavior, namely consumer behavior, even more during times of crisis. Since the COVID-19 pandemic outbreak, we witnessed a supermarket race, shelves being emptied quickly, and people making extra purchase to stock products at home.

Panic buying occurs when negative feelings like fear, panic, and feelings of uncertainty influence behavior, leading people to buy more things than usual. This type of consumer behavior is more frequently observed during periods of crisis and disruptive events, such as natural disasters, as well as public and personal health emergencies. Panic buying is not a new phenomenon reported, it already happened in other crises like SARS outbreak in China, in 2003 (Ding, 2009), and the “panic buying of salt” after the Japanese earthquake, in 2011 (Wei et al., 2011). Nevertheless, because of access to information facilitated by social media, and the universal nature of the coronavirus’ spread, panic buying became a worldwide phenomenon never seen before (Singh and Rakshit, 2020).

The pandemic outbreak is considered an alarming threat, triggering feelings of anxiety, and fear about the future, and one reason is the uncertainty about whether there will have enough food and supplies while it lasts (Kouchaki and Desai, 2015). Panic buying could be a response to the perceived lack of control regarding the future and social demands (Sim et al., 2020), and this sense of loss of control has a big impact on stress levels. In that sense, people can engage deliberately in specific types of buying behaviors in reaction to emotional distress (Sneath et al., 2009). Hence, buying more than usual can be considered as a way of coping with the feelings of uncertainty, and as an act of self-preservation.

By developing this exploratory study to create and validate an instrument to measure panic buying, this study aims to contribute with a tool to help understand the behavioral reactions of buying as a consequence of COVID-19. Appropriate psychological tools are needed to promote effective treatments and policies that provide greater awareness of human vulnerability and how people respond to threats.

Many disturbing events that occur in an individual's life can be listed – from individual crisis to collective tragedies – and their impact receives great attention in the psychological literature. However, there are fewer studies relating negative feelings and unpredictable events to purchase behaviors. This instrument contributes to the advancement of these researches, since panic buying is a behavioral phenomenon influenced by negative feelings, such as fear and panic, caused by some crisis.

Buying things as a way of coping to deal with stressful events is not new. According to Terror Management Theory, people have an internal psychological conflict resulting from a desire for self-preservation contrasted with knowledge and the certainty that death is inevitable (Harmon-Jones et al., 1997). Faced with this conflict, individuals seek to act in multiple ways to protect themselves and to find meaning, order, and stability in their world. In this sense, some studies have shown that buying things, or giving more importance to them, is a path of tension relief from existential fears (Arndt et al., 2004; Fransen et al., 2019).

The results from a search made in June 2020 in multiple databases (Web of Science, SCOPUS, EBSCO, Pubmed, and Scielo), with the term « panic buying » did not show any scale available to assess panic buying behavior. Recently, we only identified three scales that were developed during the COVID-19 pandemic, more related to the topic: Coronavirus Anxiety Scale (Lee, 2020), The Fear of COVID-19 Scale (Ahorsu et al., 2020), and COVID-19 Phobia Scale (Arpaci et al., 2020).

Nevertheless, no scale about panic buying is available until now. Furthermore, the coping outcomes caused by event-induced stress are well known, however, there are only a few studies that investigate the relation between this kind of event with consumption behavior (Sneath et al., 2009). Moreover, there are little high-quality evidences about the factors that influence panic buying (Lunn et al., 2020). Thus, all these findings led us to create the Panic Buying Scale (PBS).

The aim of this exploratory study is to develop a Panic Buying Scale (PBS) during the COVID-19 pandemic caused by the new coronavirus. Furthermore, this study aims to verify how panic buying relate to sociodemographic characteristics (gender, age, and socioeconomic class), and other psychological constructs.

## 2 Method

### 2.1 Participants

393 Brazilians took part in this study (251 women and 142 men, mean age = 42.58, SD = 14.74 years). The sample included people from all socioeconomic class, where 2.8% of respondents have declared themselves to belong to lower social class; 21.1% belong to middle-lower social class; 50.6% belong to middle class; 23.7% belong to middle-upper social class and 1.8% belong to upper social class. Of the total number of participants, 0.3% of respondents declared their level of education was elementary school degree; 7.4% high school degree; 30.5% graduation university education; 30.5% specialization degree; 17.3% master degree, and 14% PhD.

### 2.2 Instruments

It was used an online questionnaire made available on the internet. Questionnaires that had been fully answered were used. There was a section in the questionnaire which included sociodemographic questions such as gender, age, level of education, and socioeconomic class (participants positioned themselves in five options: lower, middle-lower, middle, middle-upper, upper). In addition to these questions, there were scales to assess panic buying and to the other instruments as described below.

#### 2.2.1 Panic Buying Scale (PBS)

It was applied the scale developed in this study, composed by seven items (e.g. Fear drives me to buy more than I usually do; Fear drives me to buy things to stock at home; Panic makes me buy more things than I usually do, *α* = 90). Before the items of the scale, there was the following instruction: [During the current outbreak of the COVID-19 pandemic], how would you describe your buying behavior?^1^ For each statement, we would like you to point out your degree of disagreement or agreement, considering [your recent behavior during the new coronavirus pandemic] (consider 1 = “strongly disagree” and 7 = “strongly agree”).

#### 2.2.2 Buying Impulsiveness scale

It was used a short version of the Rook and Fisher (1995) scale adapted to the Brazilian context by Marot and Lins (2018), with four items (“Just do it” describes the way I buy things; I often buy things without thinking; “I see it, I buy it” describes me; “Buy now, think about it later” describes me, *α* = .73). The scale was measured from 1 (totally disagree) to 7 (totally agree).

#### 2.2.3 Temporal focus

This measure was translated from the original (Shipp et al., 2009), and consists of 12 items assessing the amount of attention devoted to the past (e.g. I think about things from my past, *α* = .85), present (e.g. My mind is on the here and now, *α* = .75), and future (e.g. I focus on my future, *α* = .83). The items were answered on a 7-point scale (1 = totally disagree; 7 = totally agree).

#### 2.2.4 Risk perception

The 4-item scale adapted from Lins and Poeschl (2015), that asked the participants to indicate how extent they feel that the COVID-19 pandemic (new coronavirus) directly affects (1) your own life; (2) the life of your family; (3) the life of your friend and (4) Brazil (1 = not at all 7 = totally, *α* = .77).

#### 2.2.5 Life orientation test–revised (LOT-R)

It is an adaptation to the Brazilian context (Bastianello and Pacico, 2014) of the scale developed by Scheier et al. (1994). LOT-R has been used to assess individual differences in Dispositional Optimism. Three items measure optimism (e.g. In uncertain times, I usually expect the best), and three items measure pessimism (e.g. I hardly ever expect things to go my way). Respondents rate each item on a 7-point scale: 1 = strongly disagree, and **7** = strongly agree (*α* = .81).

#### 2.2.6 Need for cognition (NFC)

Need for cognition was measured using a 6-item brief version scale of Cacioppo et al. (1984), developed by Coelho et al. (2018). The translated items were taken from the NFC Brazilian version (Gouveia et al., 2015) (e.g. I would prefer complex to simple problems, I like to have the responsibility of handling a situation that requires a lot of thinking, I would prefer a task that is intellectual, difficult, and important to one that is somewhat important but does not require much thought, 1 = totally disagree; 7 = totally agree, *α* = 72).

### 2.3 Procedures

Participants were recruited by snowball sampling (Naderifar et al., 2017) using e-mails and posts on social networks. The invitations explained the survey and provided the link to access the questionnaire. In the first part of the questionnaire, a consent form was presented, showing the aim of the study, informed about the use of their data for academic and scientific purposes, and ensuring the anonymity of participants. All the ethical principles of research involving human beings were respected, according with the resolution 466/12 of the Brazilian National Health Council.

Data collection was carried out through an online questionnaire which was shared through social media networks, for 25 days, between the 10th of April and the 4th of May 2020. On April 10th, the number of deaths caused by the new coronavirus in the world exceeded 100,000, and in Brazil there were more than 1,000 confirmed deaths. In the days that followed, the world had the confirmation of more than 3,000,000 confirmed infected and more than 200,000 deaths all over the planet. On the last day of collection, May 4th, were that more than 100,000 people in Brazil had contracted the new coronavirus and more than 7,000 Brazilians died of COVID-19.

The instrument was developed in Brazilian Portuguese (see Appendix). Before applying the survey, the PBS was evaluated by three judges, who assessed the understanding of the items. After that, some terms were modified to improve the comprehension of the scale. The items were created considering the existing literature on this topic and the definition of panic buying adopted in this study. For the submission of this manuscript, the scale was translated into English by three proficient reviewers in the original language.

First, an exploratory factor analysis was performed. Subsequently, a confirmatory factor analysis was carried out using AMOS program (version 26). The estimation method used was the maximum likelihood (ML). The following indicators were used to test the goodness-of-fit of the scale: Relative chi-square (χ2/df), CFI (Comparative Fit Index), GFI (Goodness-of-Fit Index), TLI (Tucker-Lewis Index), and RMSEA (Root Mean Square Error of Approximation). Values up to 3 of relative chi-square indicator are acceptable (Iacobucci, 2010). For the indices CFI, GFI and TLI, values closer to 1 are considered adequate indicators (Garson, 2012). Lastly, for the RMSEA with a confidence interval (CI) of 90%, values up to .10 are considered acceptable (Byrne, 2010). Tests t-means difference and r's Pearson correlations were also performed.

This is an exploratory study of a barely explored concept, for this reason, to examine the convergent validity of the PBS, we included a range of variables related to different consumer behavior: Impulse Buying (Gallagher et al., 2017), Temporal Focus (Winterich and Haws, 2011), Optimism (Kakeu and Byron, 2016), Risk Perception (Sjöberg and Engelberg, 2005), and Need for Cognition (Richard and Chebat, 2016).

## 3 Results

Results indicated that the scale was appropriate for factor analysis (KMO = .92; Bartlett's test of sphericity = 1751.60, *p* < .001). The exploratory factorial analysis indicated that PBS has a unidimensional solution (Eigenvalue = 4.95), explaining 66.37% of the total variance, and showed satisfactory reliability indexes (*ω* = .92; *α* = .90; *λ* = .91), with items that had factor loading ranging from 0.60 to 0.88 (see Table 1), with skewness of 1.67 (*SE* = .12) and kurtosis of 2.74 (*SE* = 0.25).

**Table 1:** Factor Analysis of the Panic Buying Scale (PBS).

Item	*M* (*SD*)	Factor Loading
1. Fear drives me to buy things to stock at home.	2.05 (1.51)	.88
2. The fear of not having the products that I need leads me to buying more things.	1.95 (1.47)	.86
3. I panic when I think that essential products may run out from the shelves, so, that is why I prefer to buy them in bulk.	1.72 (1.32)	.82
4. Fear drives me to buy more than I usually do.	1.85 (1.38)	.79
5. Panic makes me buy more things than I usually do.	1.67 (1.30)	.77
6. One way to relieve the feeling of uncertainty is to make sure that I have a good amount of the products that I need at home.	2.48 (1.73)	.71
7. The feeling of uncertainty influences my buying habits.	2.84 (2.05)	.60
KMO		.92
Bartlett's Test of Sphericity		1751.60∗
Eingenvalue		4.65
McDonald's ω		.92
Cronbach's α		.90
Gutmann's λ		.91
Variance Explained		66.37%

*Note*: ∗*p* < .001; Extraction Method: Principal Axis Factoring.

**Figure 1:**
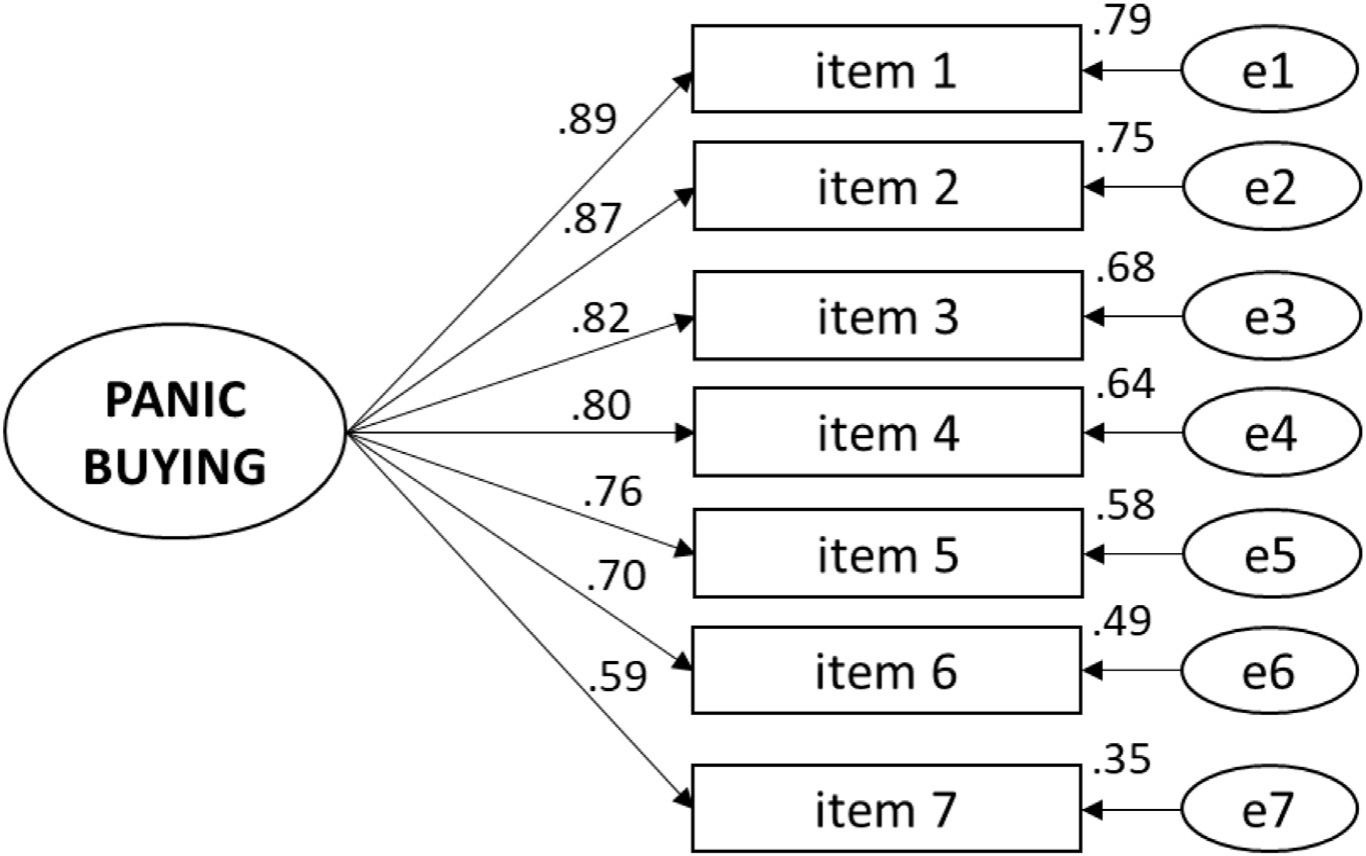
Factor structure of the Panic Buying Scale (PBS).

The results also showed a good adjustment of the proposed model: *χ*2/df = 2.83, *GFI* = .97, *CFI* = .99, *TLI* = .98, *RMSEA* = .07, CI 90% = .044–.094, (Average Variance Extracted, 61.10%, composite reliability = .92). All factor loadings were statistically significant (*p* < .001) (see Figure 1).

The results also revealed that men obtained higher levels of panic buying (*M* = 2.32, *SD* = 1.38) than women (*M* = 1.95, *SD* = 1.27), *t* (391) = 2.89, *p* = .004, *d* = 0.28, 95% IC [-0.51,0.05]. Additionally, *r* of Pearson's correlations showed that panic buying was positively correlated with impulse buying (*r* = .28, *p* < .01) past and future temporal focus (*r* = .11, *p* < .05, *r* = .18, *p* < .01), risk perception (*r* = .14, *p* < .01), and socioeconomic class perceived (*r* = .14, *p* < .01). On other hand, panic buying was negatively correlated with present temporal focus (*r* = -.16, *p* < .01), optimism (*r* = -.23, *p* < .01), and age (*r* = -.10, *p* < .05), but not with need for cognition (see Table 2).

**Table 2:** Correlation between panic buying and other constructs.

	*M* (*SD*)	1	2	3	4	5	6	7	8	9
1. Panic buying	2.08 (1.24)	1								
2. Impulse buying	1.63 (0.94)	.28∗∗	1							
3. Past focus	4.06 (1.47)	.11∗	.02	1						
4. Present focus	5.21 (1.11)	-.16∗∗	-.17∗∗	-.13∗	1					
5. Future focus	4.84 (1.32)	.18∗∗	.08	.46∗∗	-.03	1				
6. Risk perception	6.08 (0.95)	.14∗∗	-.07	.08	-.02	.12∗	1			
7. Optimism	5.53 (1.17)	-.23∗∗	-.15∗∗	-.26∗∗	.38∗∗	-.17∗∗	-.09	1		
8. Need for cognition	5.00 (1.17)	.03	-.17∗∗	-0.5	.18	-.01	.06	.30∗∗	1	
9. Age	45.58 (14.74)	-.10∗	-.02	-.22∗∗	.23∗∗	-.33∗∗	-.05	.35∗∗	.14∗∗	1
10. Socioeconomic class	3.00 (0.80)	.14∗∗	.05	-.13∗∗	.04	-.03	-.02	.20∗∗	.07	.14∗∗

∗∗*p* < .01; ∗*p* < .05.

## 4 Discussion

Our findings suggest that PBS is psychometrically acceptable in the Brazilian context, including evidence of its convergent validity. Besides satisfactory psychometric properties, PBS can be considered a good option of measure because it is not a long scale, which provokes boredom, fatigue and lack of attention, and the rate of dropout. The instrument is able to differentiate groups (e.g. gender). Additionally, PBS was positively correlated with impulse buying, past and future temporal focus, risk perception, and socioeconomic class perceived, as well as negatively correlated with present temporal focus, optimism, and age.

Although female reported higher rates of fear of COVID-19 than males (Bitan et al., 2020; Broche-Pérez et al., 2020; Zolotov et al., 2020; Sakib et al., 2020), our findings revealed that men showed higher levels of panic buying than woman. Accordingly, even though men feel less fear than women, the relationship between worry and buying is more predominant in men (Clemens et al., 2020) This results also shed light regarding the coping strategies used by gender face of fear, revealing that excessive buying could be more associate with males. Furthermore, gender differences are also seen in other kinds of consumer behavior, such as impulse buying (Aquino et al., 2020), compulsive buying (Lee and Workman, 2015), and conspicuous consumption (Segal and Podoshen, 2013; Verdugo and Ponce, 2020).

The correlation between PBS and impulse buying was the highest because both behaviors are related to emotional states. But panic buying differs from other consumer behaviors such as impulse buying, which may have different motivations and functions, and not necessarily only under the influence of negative feelings, as well as may occur in response to various contexts, involving other elements (Aquino et al., 2020). Thus, while impulse buying can be influenced by positive emotions, panic buying is always related with negative emotions as a driven consumer behavior.

Optimism was also correlated with panic buying. The negative correlation between PBS and dispositional optimism is understandable. If individuals who have more positive expectations about the future tend to be less anxious (Çikrıkci et al., 2019), then, they may engage less in panic buying. Indeed, in times of prevention by wearing masks and face shield locks, individuals with high levels of dispositional optimism could also have a “psychological shield” for cultivating more widespread positive expectations about future events.

In this sense, when the predominant temporal focus of the individual is on the past or on the future, people have a higher tendency to think and reflect on facts that have occurred, or to imagine what is coming in future days. In the present study, this amount of thought and attention dedicated to the past and future was positively correlated with panic buying. This relation indicates that thinking of nostalgia, regret, hope or worries can predict panic buying. Similarly, the risk perception about COVID-19 and how much life is affected by the pandemic relates to consideration of the future and current panic buying behaviors.

On the other hand, when analyzing the predominant time focus on the present, it can be observed that it correlates negatively with panic buying. This inverse relationship indicates that focusing on the here and now, and keeping the mind on the present day, can also protect people from dysfunctional behavior.

As the number of adversities due to COVID-19 remains to increase by a great amount in Jul 2020, individuals continue to suffer from high levels of anxiety and fear for an extended period (Lee, 2020), even more with the threat that new waves of infections could occur. The first moment of fear is usually more intense, while the situation is completely adverse and overwhelming.

The advancement of the pandemic teaches us that it is essential that we do not ignore the psychological impact that COVID-19 has on individuals and society. While the COVID-19 crisis continues to progress in Brazil^2^ and in many countries, this exploratory study, demonstrating the reliability and validity of PBS, offers a useful tool that can be applied to reach other populations, contexts and applications.

Because a pandemic is a threat that causes fear, anxiety, and uncertainty, it also raises concerns about whether there will be sufficient supplies or about how long economic instabilities will last. The excessive buying and storage behaviors can cause demands that overcome the supply, generating product shortages in the middle of the crisis, or even triggering a herd panic buying, increasing feelings of instability and anxiety.

Socio-psychological considerations that address what and why the behaviors of individuals shown during the coronavirus crisis will be important studies in the future to provide data that manage public decisions that affect individuals and society. Consumers, healthcare professionals and public decision makers need to be aware of the potential impact of these negative feelings on purchasing behavior, as this helps to develop interventions that prevent or minimize emotional and economic distress in the midst of crises. The understanding of purchasing decisions influenced by drastic changes in the environment can pave the way to reduce and combat post-traumatic disorders in times of crisis, or to combat the effects of pandemic-related fear.

Indeed, panic buying is more commonly observed during periods of crisis and disruptive events, but not only in collective event-induced stress but also in personal crisis. There are many possible causes for panic buying, besides a pandemic. Researches about panic buying will have a greater long-term impact if it can extend the applicability beyond an outbreak. For this reason, the items of PBS were developed to be also used in a period without crisis, and the instructions of the scale can be adapted for other extreme social or individual situations.

Personal disruptive events could be also related to panic buying, and to psychological disorders, which panic and fear are related symptoms (e.g. post-traumatic stress disorder, anxiety disorders, panic disorder, social phobia, agoraphobia), and other types of buying behavior disorder (e.g. compulsive buying). Furthermore, it is probable that the link between psychological disorders and panic buying can be stronger during stressful periods.

This study has some limitations. We highlight the sample collected, which does not represent the majority of the Brazilian population in socioeconomic class or education level. According to the survey “Education at Glance”, produced by the Organization for Economic Co-operation and Development (OECD, 2019), only 21% of Brazilians between 25 and 34 years of age have completed higher education. The highly educated characteristic of the participants in this study (61.8% of people with post-graduation) may indicate a bias of this study, as it does not accurately reflect the reality of most Brazilians.

Another limitation is the strength of the correlations. Indeed, the size of the correlations can be considered small (see for example, Cohen, 1988). Nevertheless, because it was an exploratory study, significant correlations between PBS and the variables under study add to the set of evidence of validity and reliability and allow attesting to the instrument's validity.

Future investigations should focus on these aspects and investigate other variables that were not included in this study such as personality traits, media exposure, and social influence (e.g. herd behavior). New studies with this instrument could provide more evidences of validity and verify if the instrument is applied in other countries. Thus, cross-cultural studies are necessary to advance and expand our knowledge about panic buying, to improve an instrument that can be extremely useful to understand the psychosocial phenomena associated with consumer behavior. Even in Brazil, with an enormous potential for research, considering its ethnic, religious, and social diversity.

If the pandemic is not completely under control, there may be new peaks of panic buying, and studies conducted throughout a stressful event may bring new findings about this. That is why longitudinal studies are recommendable to be carried out, to assess the evolution of panic buying, trying to link to economic indexes, news, and death toll, as well as making a comparison of panic buying between, during, and after pandemic. Moreover, future studies could explore types of purchases made at times of panic. We assume that the choice is higher for essential products, so we expect that more researches may add this information.

Finally, besides focusing on the antecedents of panic buying behavior, investigations should also identify the consequences and impact of this type of consumer behavior in the retail market, family financial problems, and mental health. Only a body of empirical research will provide some findings and cues for public policymakers and health professionals, avoiding shortcuts in the retail market, and creating strategies for mental health promotion.

## Declarations

### Author contribution statement

S. Lins, S. Aquino: Conceived and designed the experiments; Performed the experiments; Analyzed and interpreted the data; Contributed reagents, materials, analysis tools or data; Wrote the paper.

### Funding statement

This research did not receive any specific grant from funding agencies in the public, commercial, or not-for-profit sectors.

### Competing interest statement

The authors declare no conflict of interest.

### Additional information

No additional information is available for this paper.

## Appendix Portuguese Brazilian version of the Panic Buying Scale (PBS)

**Durante o atual avanço da pandemia de COVID-19,** como tem sido o seu comportamento de compra? Para cada afirmação, gostaríamos que você apontasse seu grau de DISCORDÂNCIA ou de CONCORDÂNCIA, considerando seu comportamento **recente durante a pandemia do novo coronavírus.** (considere que 1 = “Discordo totalmente” e 7 = “Concordo totalmente”)

1. O medo me leva a comprar coisas para ter estoque em casa.
2. O medo de não ter produtos que eu preciso me leva a comprar mais coisas.
3. Eu fico em pânico ao pensar que produtos essenciais podem faltar nas prateleiras, por isso que eu prefiro comprá-los em grande quantidade.
4. O medo me leva a comprar mais do que o normal.
5. O pânico me faz comprar mais coisas do que eu costumo comprar
6. Uma forma de aliviar o sentimento de incerteza é garantir que tenho em casa uma boa quantidade produtos que eu preciso.
7. O sentimento de incerteza influencia os meus hábitos de compra.

Obs. No enunciado da escala, o que está destacado em **negrito** deve ser alterado para o contexto estudado conforme necessidade do pesquisador.

## Footnotes

1 In the scale's instructions, what is in brackets should be changed for the context studied, according to the researcher's needs.

2 In Brazil, there have been 3.224.876 confirmed cases of COVID-19 with 105.463 deaths, until August 15th 2020, according to official data released by World Health Organization (WHO) in https://covid19.who.int/region/amro/country/br.
